# γδ TCR T lymphoblastic leukemia in a child presenting with marked hyperleukocytosis

**DOI:** 10.1002/ccr3.4472

**Published:** 2021-07-21

**Authors:** Jirong Mass, Bachir Alobeid

**Affiliations:** ^1^ Department of Pathology and Cell Biology Columbia University Medical Center New York NY USA

**Keywords:** children, hyperleukocytosis, leukemia, lymphoblastic, lymphoma, pediatric, T cell, γδ TCR

## Abstract

γδ TCR T lymphoblastic leukemia is rare in children and should be differentiated mainly from hepatosplenic T‐cell lymphoma in this age group.

## Graphical abstract

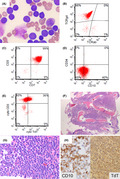

Here, we report a rare case of pediatric γδ TCR T lymphoblastic leukemia with unusual presentation of marked hyperleukocytosis. We also describe the distinct features that helped exclude other γδ TCR T‐cell neoplasms, focusing on hepatosplenic T‐cell lymphoma which is the main differential diagnosis that requires different management and therapy.

A 30‐month‐old female patient presented with marked hyperleukocytosis (WBC: 869 × 10^9^/L; N: 4.5–11.0 × 10^9^/L) and 95% circulating blasts (Figure 1, Panel A). Cervical lymphadenopathy was detected but no hepatosplenomegaly. Peripheral blood (PB) flow cytometry (FC) detected a major immature T‐cell population: CD45+, CD3+, γδ TCR+, CD5+, CD10+/partial, TdT+/partial, CD34−, CD1a−, CD4−, and CD8− (Figure 1, Panels B–E). The bone marrow (BM) was diffusely infiltrated by similar blasts (Figure 1, Panels F–H). Complex clonal cytogenetic abnormalities with multiple sub‐clones were detected but no 7q or 8 chromosomal abnormalities. Deletions of CDKN2A, CDKN2B, and MTAP were detected by SNP Oligonucleotide microarray. PCR of the TCR beta gene was polyclonal. Overall findings were supportive of T lymphoblastic leukemia with γδ TCR phenotype (γδ TCR T‐ALL). The patient is currently in remission after her third induction therapy.

**FIGURE 1:**
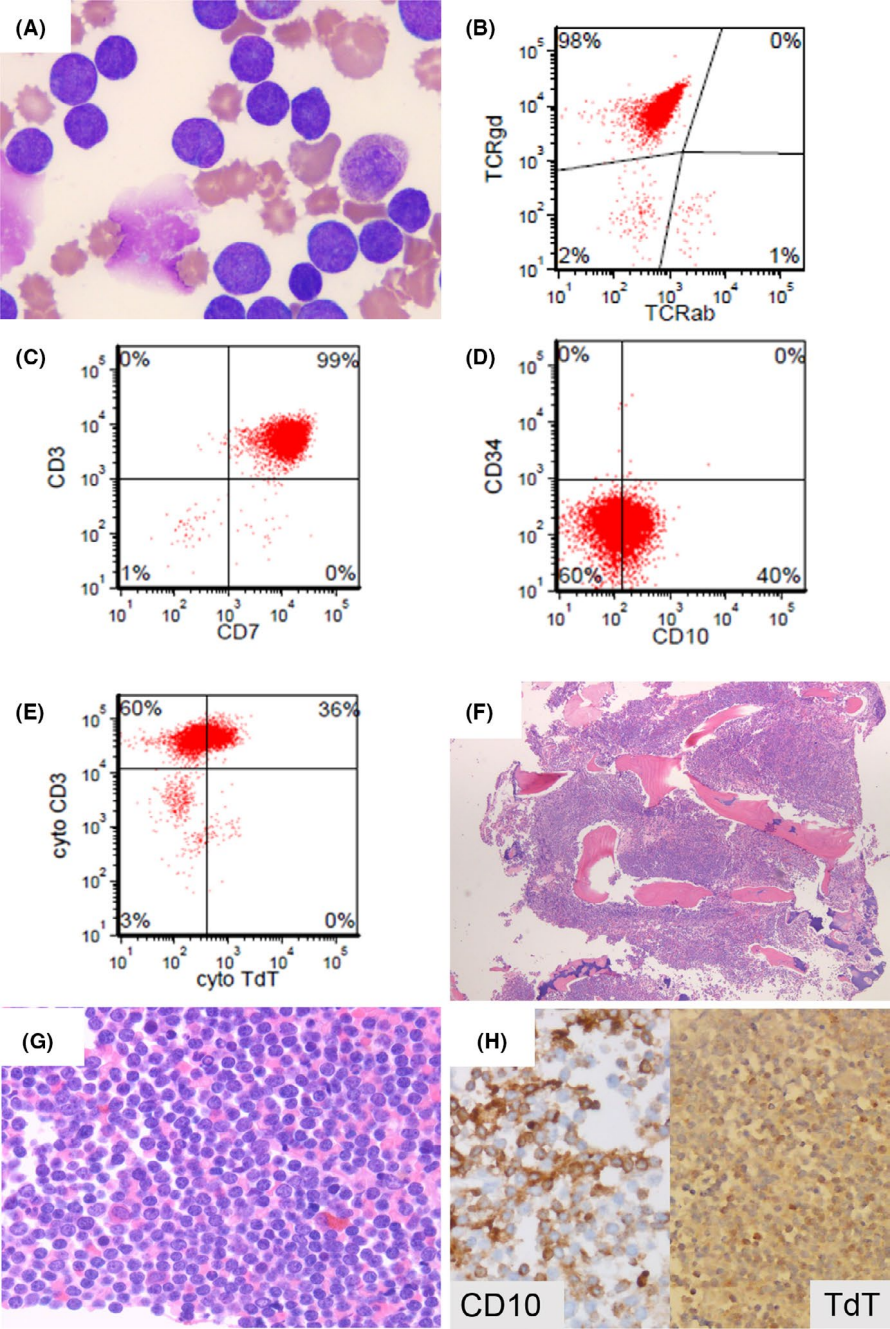
The peripheral blood smear shows numerous blasts (panel A; 100× objective; Wright‐Giemsa stain) with aberrant, immature γδ TCR T‐cell immunophenotype detected by flow cytometry analysis (panels B–E). The bone marrow biopsy shows extensive, diffuse involvement by similar blasts (panels F and G; 4× and 40× objectives, respectively; H&E stain). The blasts are positive for CD10 and TdT (panel H; 40× and 20× objectives, respectively; Immunohistochemistry)

The main differential diagnosis in this case is hepatosplenic T‐cell lymphoma (HSTCL). The features that helped exclude HSTCL include the marked hyperleukocytosis, blast cell morphology, cervical lymphadenopathy with absence of hepatosplenomegaly, diffuse BM infiltration, expression of CD5 and immaturity markers, absence of isochromosome 7q and trisomy 8, and deletions of CDKN2A, CDKN2B, and MTAP which are typical of T‐ALL.^1^ Children with γδ TCR T‐ALL may have less favorable outcomes than other T‐ALL patients, which might require more intensive upfront treatment.^2^

## CONFLICT OF INTEREST

None declared.

## AUTHOR CONTRIBUTION

JM and BA involved in conception and design, manuscript preparation.

## ETHICAL APPROVAL

The anonymity and confidentiality of the patient were preserved by not revealing the name and identity in the case report.

## Data Availability

Data sharing not applicable to this article as no datasets were generated or analysed during the current study.
